# Airborne and Terrestrial Observations of the Thermal Environment of Urban Areas Surrounding a High-Rise Building during the Japanese Winter

**DOI:** 10.3390/s20020517

**Published:** 2020-01-16

**Authors:** Haruki Oshio, Kan Chen, Takashi Asawa

**Affiliations:** 1 School of Environment and Society, Tokyo Institute of Technology, 4259-G5-2, Nagatsuta-cho, Midori-ku, Yokohama, Kanagawa 226-8502, Japan; oshio.haruki@nies.go.jp (H.O.); soryuw@foxmail.com (K.C.); 2 Center for Global Environmental Research, National Institute for Environmental Studies, Tsukuba, Ibaraki 305-8506, Japan; 3 Tongji Architectural Design (Group) Co., Ltd., No. 1230, Siping Road, Shanghai 200092, China

**Keywords:** urban thermal environment, air temperature, high-rise building, mobile observation, thermal remote sensing

## Abstract

We investigated the distribution of air temperature (T_a_) and the factors affecting it in low-rise areas surrounding an isolated high-rise building during the Japanese winter. The study site was the central part of a regional city in Japan (36°5′ N, 140°12′ E), lying north-east of the Tokyo metropolitan area. The daytime surface temperature (T_s_) in the shade is generally considered to be comparable to T_a_; however, according to airborne remote sensing conducted in December 2009 where a multi-spectral scanner was installed on a fixed-wing aircraft, T_s_ for pavements in the shade of a high-rise building was significantly lower than T_a_ of sub-urban areas, indicating an influence of cold storage on T_s_. Then, we conducted mobile observations using instruments (thermocouple, four component radiometer, and so on) installed on a bicycle in January 2016 to investigate the detailed distribution of T_a_ and the factors affecting it. The results showed the T_a_ over the pavements in the shade of the high-rise building was lower than the T_a_ of sunlit areas in the same urban area by −2 °C and lower than the T_a_ of sub-urban areas by −1–1.5 °C, although the advection effect was large due to strong winds around the building. In conclusion, a locally lower T_a_ compared to the surrounding areas can develop during the day in winter, even in spaces that are open to areas beyond the canopy.

## 1. Introduction

Recently, there has been a global increase in the construction of high-rise buildings throughout urbanized areas. Assessing their environmental impact is important for creating comfortable and environmentally-friendly urban areas. In terms of thermal environment, a typical problem in urban areas is urban heat islands (UHIs), areas that have a higher air temperature (T_a_) than the surrounding sub-urban areas. The primary factor for UHIs is the difference in surface materials between urban and sub-urban areas, and many studies have reported daytime and nighttime UHIs [1,2]. However, some studies have also reported urban cool islands (UCIs), which are areas that have a lower T_a_ than the surrounding areas. These UCIs have been primarily observed in the daytime for densely built central areas with high-rise buildings [3,4,5,6]. The primary cause is considered to be interception of the incoming solar radiation on the inner part of the canopy by the buildings if the advection effect is small [3,4,5,6]. This is a similar situation to deep canyons (the height of the buildings is several times greater than the width of the road), where lower T_a_ inside the canyon compared to outside of the canyon have been observed [7,8,9]. The difference in thermal inertia between urban and sub-urban areas also seems to be an important factor for UCIs [3,4,5,6]. Therefore, the formation of UCIs largely depends on spatial geometries of urban canopies.

In Japan, in recent years, high-rise apartments are frequently constructed as part of the redevelopment of a part of an old town due to their designation as efficient utilization district (i.e., districts where regulations regarding building height are less strict) by the government. Therefore, it is common to see an isolated high-rise building adjacent to low-rise areas, especially in regional cities. We assume that the influence of a high-rise building on the thermal environment of surrounding areas becomes particularly significant in winter because of the high-rise building casting a shadow over a large area due to the lower solar altitude. Long-time interception of direct solar radiation by a high-rise building seems to be a factor for locally low T_a_, similar to the UCIs mentioned above. However, the situation is different from densely built central areas and deep canyons: in the case of the shade of a high-rise building, certain spaces are open to areas beyond the canopy, and the extent of the shade is limited. Although many studies have investigated the relationship between T_a_ and urban spatial geometry [10,11,12,13,14], the overall effect of an isolated high-rise building on the surrounding T_a_ is not yet completely clear. Because coldness causes uncomfortable urban spaces and increased heating energy needs in buildings during winter, investigating the distribution of T_a_ around a high-rise building during winter from a point of view of UCIs is essential.

This study aims to investigate the actual distribution of T_a_ and the factors affecting it in low-rise areas surrounding an isolated high-rise building during the Japanese winter. The study site is central part of Tsuchiura city (36°5′ N, 140°12′ E), a regional city in Japan, where a high-rise apartment building was built as part of the redevelopment of a low-rise urban district. First, the distribution of surface temperature (T_s_), a primary factor influencing T_a_ [15,16], was investigated by airborne thermal remote sensing. The remote sensing observation was conducted in December 2009 using a multi-spectral scanner (MSS) installed on a fixed-wing aircraft. Although lower T_s_ in shaded areas than in sunlit areas are obvious, we investigated quantitative differences in T_s_ within the shaded areas according to surface materials and spatial geometry. Subsequently, bicycle-based mobile observations were conducted in January 2016 to obtain the detailed distributions of T_a_ and associated factors (i.e., T_s_, wind velocity, and radiation budget). Thermocouple, humidity sensor, handheld hot-wire anemometer, and four component radiometer were installed on a bicycle. The target and route of mobile observations were decided based on the results of remote sensing, and the relationship between T_a_ and the associated factors was investigated. There was a time interval of about 6 years between the remote sensing and the mobile observations; therefore, we used these data carefully so that the conclusion of the present study was not affected by the time interval.

## 2. Study Site

**Figure 1:**
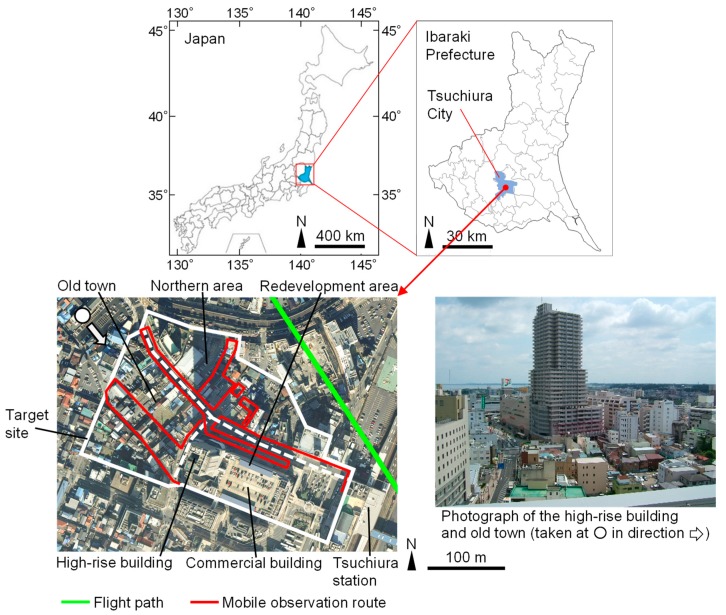
Location of the study site (**top**) and photographs of the study site (**bottom**), including the flight path of remote sensing and the mobile observation route.

Our study site is located in the central part of Tsuchiura city, Japan (36°5′ N, 140°12′ E). Tsuchiura lies north-east of Tokyo metropolitan area and is a typical Japanese regional city. The study site was selected as an example of a high-rise apartment building that was built as part of the redevelopment of a low-rise urban district. The location of our site together with aerial photographs and photographs taken at the site are shown in Figure 1. The site is located west of Tsuchiura railway station and consists of a redevelopment area, a northern area, and the old town, with all three areas adjacent to one another. A 31-story apartment building with a height of 109 m is located in the redevelopment area of our site and this area also includes a large commercial building. The northern area of our study site mainly consists of medium- or small- scale commercial and residential buildings made of reinforced concrete (RC), while the old town area of the site consists mainly of low-rise wooden detached houses.

## 3. Airborne Thermal Remote Sensing

### 3.1. Data Collection and Processing

Specifications of the remote sensing observations are shown in Table 1. These observations were conducted on a sunny winter’s day (22 December 2009) both during the day and after sunset. Daytime observations were performed to investigate the T_s_ characteristics in the shade of the high-rise building. Post-sunset observations were done to confirm the influence of daytime shade on the distribution of T_s_ later in the day. A MSS installed on a fixed-wing aircraft (AZM, Nakanihon Air Service, Nagoya, Japan) was used and the flight path is shown in Figure 1. A flight altitude of 500 m was used, yielding a spatial resolution of 0.63 m for nadir viewing. Geometric correction was first conducted (Appendix A). Subsequently, the obtained radiance (directional radiometric brightness) of the thermal infrared band used (Table 1) was converted to brightness temperature using a scanner-specific equation. The accuracy of the scanner itself for brightness temperature measurement was 0.3 °C. The brightness temperature was converted to T_s_ by conducting atmospheric correction and emissivity correction. The details of the conversion are described in Appendix B and Appendix C. Pixels of roofs with low-emissivity materials were manually identified and excluded from the analysis (i.e., masked in T_s_ images shown in Section 3.2.1 and Section 3.2.2, and not used for the histogram in Section 3.2.3). This is because the large difference between the actual and the assumed emissivity (a constant value was used as described in Appendix C) yields a significant difference between the actual T_s_ and the calculated T_s_ for such materials. The manual identification was conducted according to the pixel value (T_s_), the visible image acquired by the MSS, and the results of an in-situ survey.

**Figure 2:**
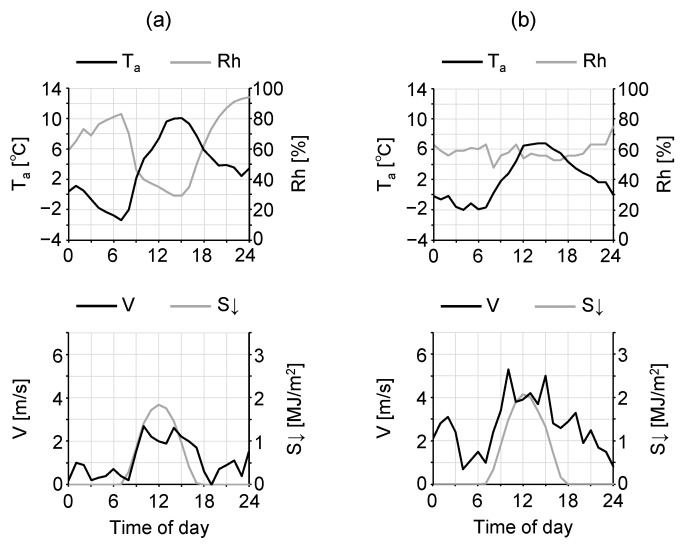
Meteorological conditions of the observation days derived by AMeDAS Tsuchiura site (air temperature (T_a_) and wind velocity (V)) and AMeDAS Tsukuba site (relative humidity (Rh) and downward short-wave radiation (S↓)): (**a**) remote sensing and (**b**) mobile observations.

The meteorological conditions of the observation day are shown in Figure 2a. The observations were performed on a clear-sky day (cloud cover of about 10%). Both T_a_ and wind velocity (V) were derived from the Japan Meteorological Agency (JMA) Automated Meteorological Data Acquisition System (AMeDAS) observational site in Tsuchiura city, 2 km north of the study site (36°6′ N, 140°12′ E). Relative humidity (Rh) and downward short-wave radiation (S↓) were not observed at the Tsuchiura site but were derived from the AMeDAS Tsukuba observational site at 7.5 km west of the study site (36°3′ N, 140°7′ E). Consequently, these AMeDAS data were used as representative meteorological data for the study site and is described with a prime symbol as T_a_′.

### 3.2. Distribution of T_s_ over the Study Site

#### 3.2.1. Daytime

**Figure 3:**
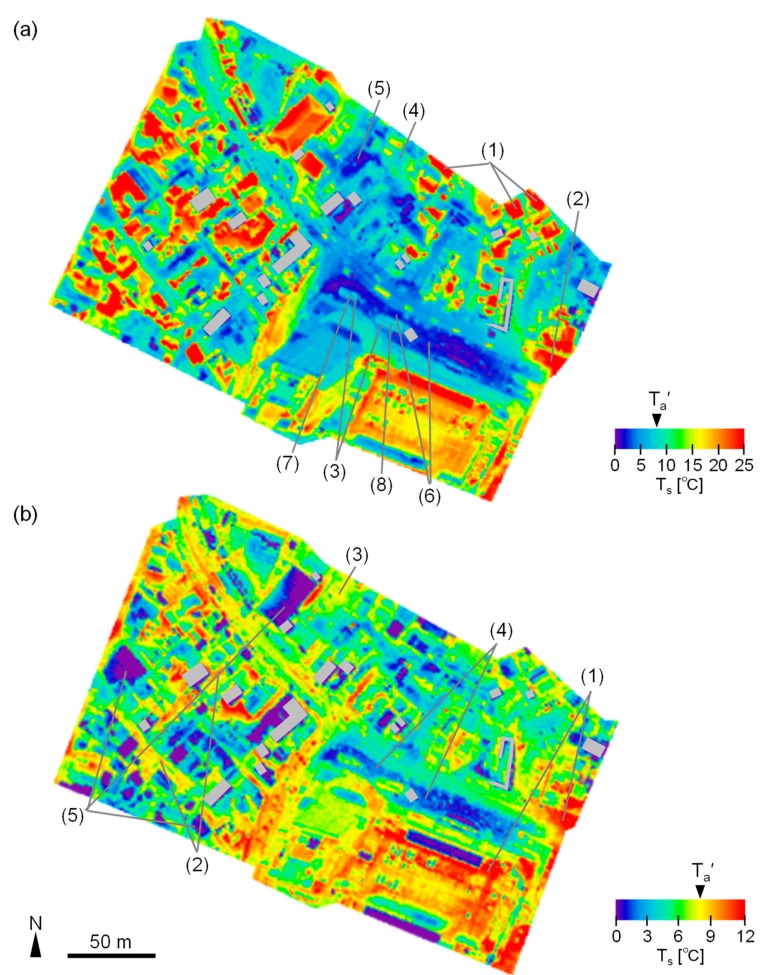
Distribution of remotely sensed T_s_: (**a**) daytime and (**b**) after sunset. The numbers in the figure indicate specific points discussed in the main text. Representative air temperature (T_a_′) is depicted for each time period. Gray masks indicate materials with low emissivity where there is a possibility that the calculated T_s_ differs significantly from the actual T_s_.

The distributions of T_s_ for daytime and post-sunset are shown in Figure 3a,b, respectively. As shown in Figure 3a(1) the T_s_ of roof surfaces reached 30 °C and the T_s_ of asphalt pavements was about 25 °C in full sun since the morning (Figure 3a(2)). T_s_ was comparable to T_a_′ (8.1 °C) for short plants in the shade (Figure 3a(3)). On the north side of the high-rise building, T_s_ was equal to or lower than T_a_′ even on the roofs (Figure 3a(4)) and pavements (Figure 3a(5)). The T_s_ of the pavement in front of the high-rise building and the adjacent commercial building was 2–3 °C when T_a_′ was 8.1 °C (Figure 3a(6)). The difference in T_s_ between the pavement and short plants, soil, and roof (Figure 3a(3), (7), and (8), respectively) is conspicuous. Since differences in the radiation budget (i.e., S↓ + L↓ in daytime and L↓ nighttime) between these materials were considered to be minimal, this result indicates that cold storage of the pavement having high heat capacity strongly affected the significantly lower T_s_ than T_a_′.

#### 3.2.2. Post-Sunset

Asphalt pavement that had been in the sun for long time showed the highest T_s_ (12 °C, Figure 3b(1)), when T_a_′ was 7.9 °C. Although there were streets and parking lots where T_s_ was somewhat higher than T_a_′ in the old town (Figure 3b(2)), few areas showed such high T_s_ values in the northern area and the T_s_ value was low even for the parking lot with no multi-story buildings around it (Figure 3b(3)). Regarding the front of the high-rise building and the adjacent commercial building, T_s_ was 1–2 °C (Figure 3b(4)) when T_a_′ was 7.9 °C. The result that low T_s_ last after sunset further indicates the contribution of cold storage of the pavement. The lowest T_s_ was also observed for roofs with a low heat capacity (Figure 3b(5)).

#### 3.2.3. Difference between the Northern Area and the Old Town Area

**Figure 4:**
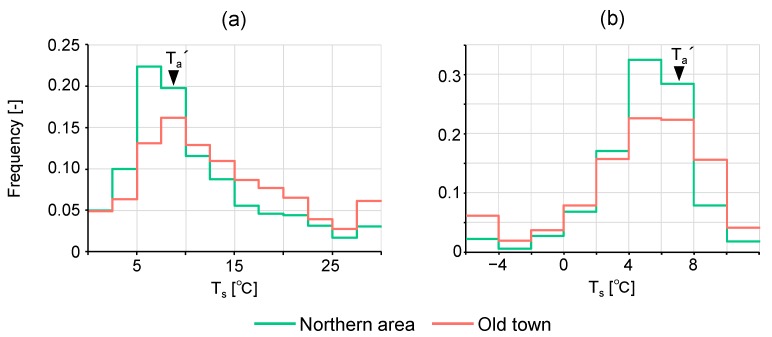
Histogram of remotely sensed T_s_ for the northern area and the old town: (**a**) daytime and (**b**) after sunset. Representative air temperature (T_a_′) is depicted for each time period.

To confirm the aforementioned characteristics, especially significantly low T_s_ of the pavement in front of the high-rise building, quantitatively, a histogram of T_s_ was derived for the northern area and the old town (Figure 4). In the daytime investigation, the difference is conspicuous. The northern area shows a higher frequency of pixels with low T_s_ compared to the old town. The peak of frequency of the northern area corresponds to a T_s_ of 5–10 °C, which is comparable to T_a_′ and seems to consist of shaded areas. A higher frequency of 2.5–5 °C is also remarkable, being caused by cold storage in pavement with a high heat capacity that was in the shade for a long time, as observed in the front of the high-rise building. After sunset, for the old town, it is considered that the presence of a large number of streets and parking lots in the sun contributed to the frequency of high T_s_, and a significantly low T_s_ for the roofs of wooden houses contributed to the frequency of low T_s_. Due to the many parking lots and RC buildings in the northern area, T_s_ is generally expected to be high. However, the frequency of T_s_ ≥ 8 °C is lower than that for the old town. Of course, shade from the high-rise building during the day contributed to this result; however, it is important to note that cold storage of materials having high heat capacity such as pavement appears to suppress the increase of T_s_. Although ortho-correction using building height was not conducted, differences in the shape of the histogram of T_s_ between the two areas is barely explained by the difference in viewing angle (the viewing zenith angle differs between areas as shown in Figure 1). Details are described in Appendix A.

## 4. Mobile Observations

### 4.1. Data Collection

**Figure 5:**
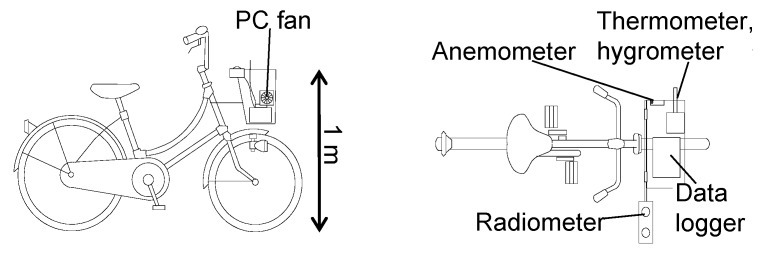
Schematic diagram of devices installed on a bicycle for mobile observations.

Mobile observations were carried out on a clear winter’s day (20 January 2016). There is a time difference of about 6 years between the remote sensing observations and the mobile observations, but the land cover and spatial geometry hardly changed in the study site (only a few buildings changed to open spaces). Therefore, the mobile observations were conducted to confirm the detailed characteristics of T_a_ and associated factors for areas where significantly low T_s_ was observed by remote sensing. The observation route is shown in Figure 1. Note that the remotely sensed T_s_ is not directly compared with the mobile observation results in the following sections. However, these data can be used complementary to understand the abovementioned phenomena. Observation items and devices used are shown in Table 2. Observations included T_a_, Rh, V, and downward and upward short-wave radiation (S↓, S↑) as well as long-wave radiation (L↓, L↑). L↑ was used as a proxy of T_s_, and relationship between areas with low T_s_ and distribution of T_a_ was discussed by investigating the distribution of L↑ and T_a_. A T-type thermocouple (Ø 0.1 mm) was installed in a forced ventilation pipe on the carrier basket of a bicycle to observe T_a_ (Figure 5), and the observer walked whilst pushing the bicycle at a uniform speed. The observer stood on the left side of the bicycle so that a view factor of the observer from the radiometer was minimal and care was taken to ensure that the radiometer was not in the shadow of the observer. The view factor of the bicycle and the influence of shade of the bicycle on the radiometer were also minimal.

Data were recorded every 2 s, with the time constant of the radiometer being 17 s and 18 s for short-wave and long-wave radiation, respectively. The observer’s walking speed was approximately 1 m/s, indicating that moving 18 m under the same radiation condition as a target point was required. However, the boundary between sunlit and shaded area estimated from the observed data differed from the actual boundary by only few meters. The time constants of the humidity sensor and anemometer were 1 s and 4 s, respectively. Therefore, the influence of the time constant on the obtained data was minimal. Although the obtained V was relative velocity, the distribution of the actual velocity could be discussed using this data as the walking speed was low enough compared to the range of V along the observation route (order of several m/s).

Observations were carried out by two persons; one pushed the bicycle and the other recorded the times when checkpoints were passed. Checkpoints were set at intervals of 10–100 m; road corners, the start or end of pedestrian crossings, and the front entrances of buildings were used as checkpoints. Target times were 10:30, 12:00, 15:00, 17:00, and 19:00 and each observation took approximately 30 min to record (target time ± 15 min). The influence of variations in background meteorological conditions during observations was minimal, as meteorological conditions were stable throughout the day. The location information of each data point was determined using the passage time and location information of the checkpoints as well as the recording time.

### 4.2. Results

**Figure 6:**
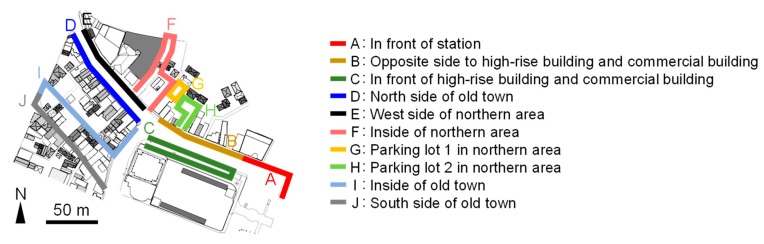
Division of mobile observation route into sections.

**Figure 7:**
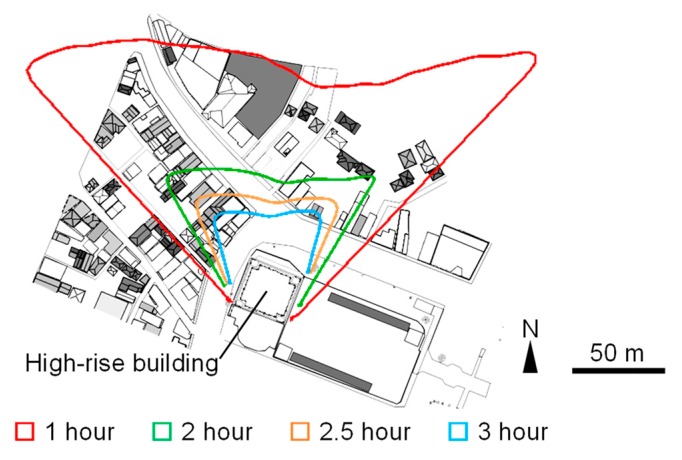
Shadow time diagram of the high-rise building for winter solstice.

**Figure 8:**
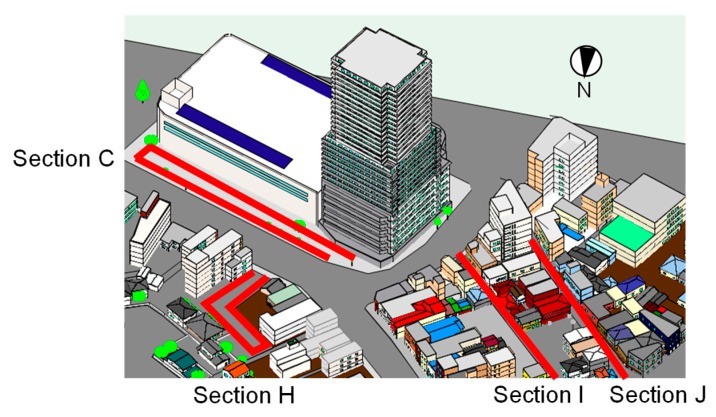
3-D CAD model of the target spaces.

Meteorological conditions of the observation day derived by AMeDAS are shown in Figure 2b. The daily highest temperature was 3.5 °C lower than that of the remote sensing observation day, and the wind velocity was higher. The daily highest temperature and wind velocity were also lower and higher, respectively, than the average values in Tsuchiura city in January (approximately 9.0 °C and 2.0 m/s). However, conditions on this day were considered those of a typical winter’s day, allowing for a discussion of the winter T_a_, V, and radiation budget. Characteristics of these quantities were expected to vary among areas in the site. Therefore, the observation route was divided into sections as shown in Figure 6 and an analysis was conducted considering a shadow time diagram of the high-rise building (Figure 7). The diagram was derived from a 3-D model (generated based on GIS data and in situ observations [17]) of the high-rise building, generated in computer aided design (CAD) software and shows the amount of time for which the high-rise building casts its shadow on each point during the winter solstice. The 3-D CAD model of the target spaces are shown in Figure 8.

**Figure 9:**
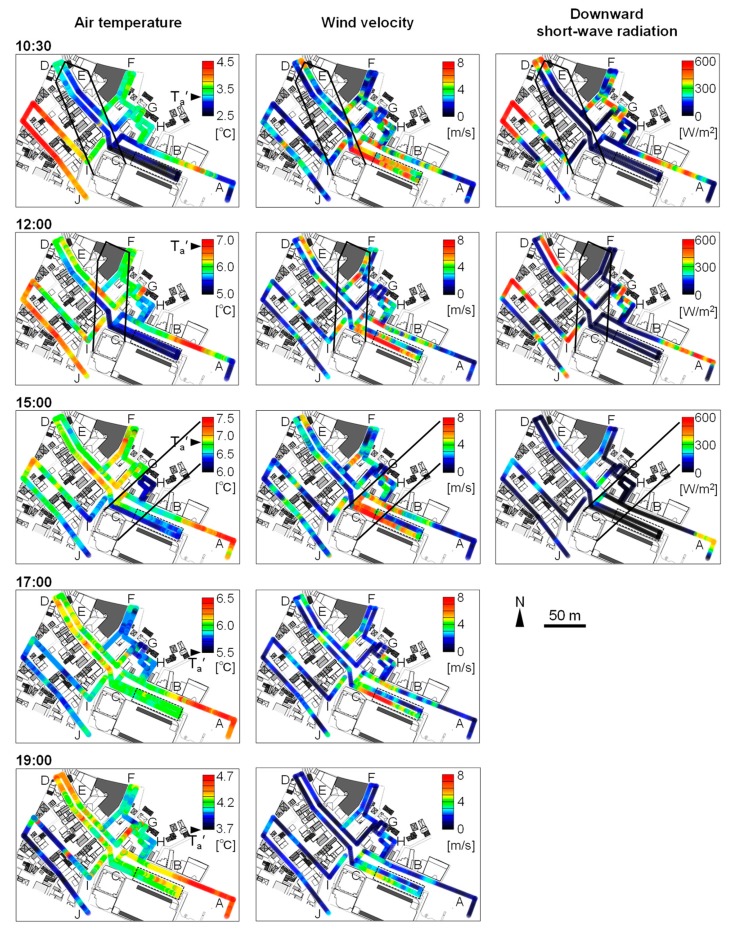

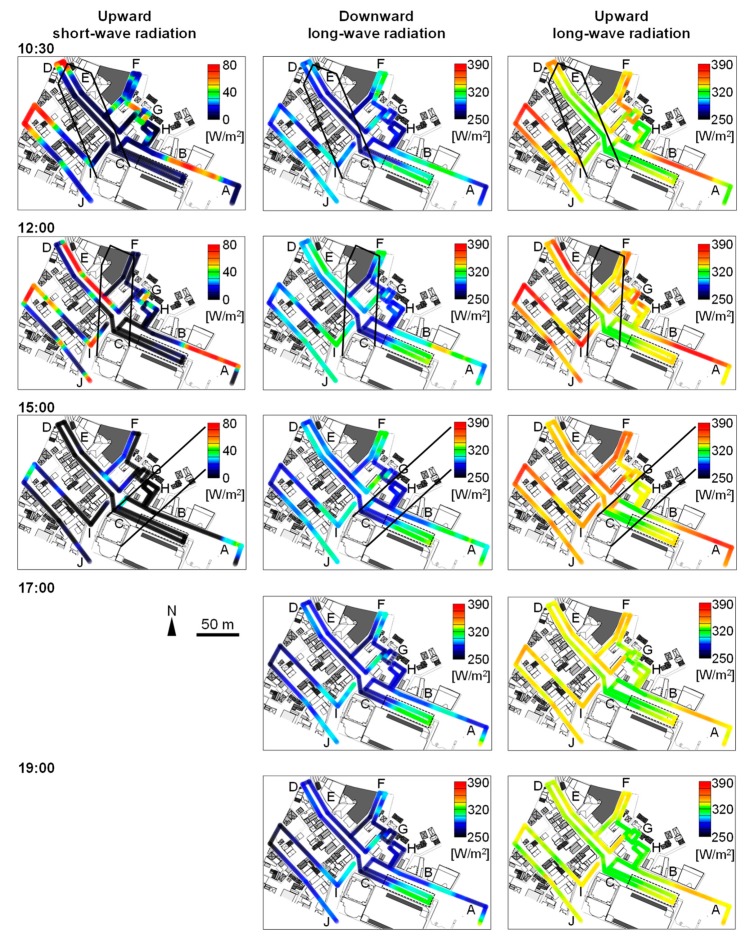
Results of mobile observations: the black line indicates the shadow diagram of the high-rise building whilst the broken line represents a large roof constructed in front of the commercial building. Names of the sections are depicted by A–J. Representative air temperature (T_a_′) is depicted for each time period.

**Figure 10:**
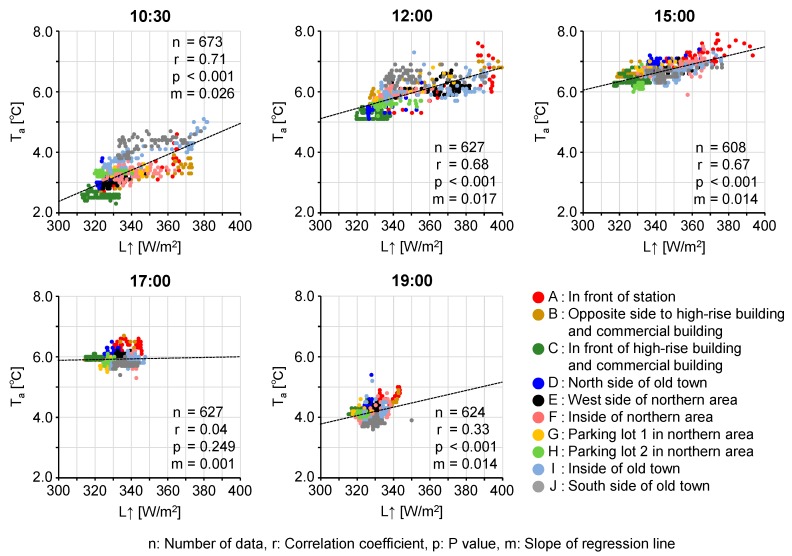
Scatter-plots of upward long-wave radiation (L↑) and air temperature (T_a_) derived from mobile observation.

Shadow diagrams of the high-rise building, generated from the 3-D CAD model, and observation results are presented in Figure 9. As expected, S↓ and L↑ differ significantly between observation points, and the points showing large (small) S↓ correspond to those showing large (small) L↑. In addition, locality of T_a_ was also observed: differences in T_a_ between observation points reached about 2 °C during daytime. T_a_ of Section C (in front of the high-rise building and the adjacent commercial building) was lower than T_a_′ during daytime. L↓ in the east part of section C was relatively high throughout the observation period as the sidewalk in front of the commercial building was covered by a large roof (broken line in Figure 9). This roof was made of transparent material and had not been constructed when the remote sensing observation was made. Scatterplots of L↑ and T_a_ are shown in Figure 10.

Each point in the plot represents each recorded data set. The distribution range on the plot clearly differs between sections. There is a relatively high positive correlation between L↑ and T_a_ in the daytime, indicating that T_a_ is low in areas with low T_s_. Especially, both L↑ and T_a_ are low during the day in Section C.

**Figure 11:**
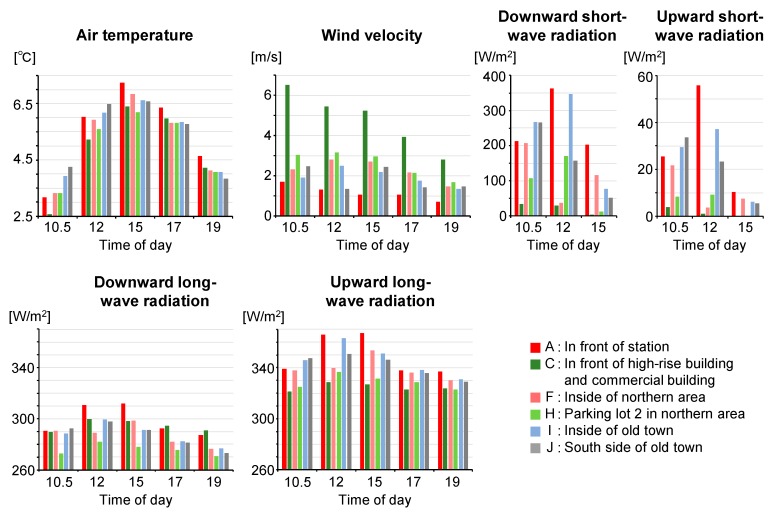
Daily variations of mobile observation results averaged over each section.

The time series of the observed values averaged over each section are shown in Figure 11. Six characteristic sections are presented. For Section A (in front of the station), where the ground is covered by asphalt pavement and no south-facing buildings, $\bar{S↓}$ increased at and after 12:00, and $\bar{L↑}$ and $\bar{T_{a}}$ (spatially averaged values) were high throughout the afternoon. For Section C, high values of $\bar{V}$ throughout the observation period indicate the occurrence of building wind (strong wind around a high-rise building). Both $\bar{S↓}$ and $\bar{L↑}$ were the lowest in this section throughout the day, while $\bar{T_{a}}$ was lowest at 10:30 and 12:00. As shown in Figure 9, L↑, which corresponds to T_s_, was greater under the large roof than outside of it, indicating that atmospheric radiative cooling is primarily responsible for the formation of low T_s_, as the roof appeared opaque to long-wave radiation. However, the difference in T_a_ under the roof and outside of it was small. In contrast to the daytime, at 19:00, $\bar{T_{a}}$ was not low compared to other sections. Regarding Section F (inside the northern area), $\bar{S↓}$ was low at 12:00. It is inferred from the distribution of S↓ in this section that the high-rise building cast its shadow on an area 130 m away. This corresponded well to the shadow diagram (black line in Figure 9). $\bar{L↑}$ and $\bar{T_{a}}$ were the third lowest following the street adjacent to the high-rise building (section C) and in the parking lot surrounded by buildings (Section H). Although the shadow time was about 1 hour (Figure 7), $\bar{T_{a}}$ was low. For Section H (parking lot 2 in the northern area), $\bar{S↓}$ was low throughout the daytime as this section was shaded by the building on the eastern side of the section (Figure 8) in the morning and by the high-rise building around noon (Figure 9). Both $\bar{L↑}$ and $\bar{T_{a}}$ were consistently low for the observation period. The occurrence of detached houses around this section indicates that a severe thermal environment was formed even in living spaces 70 m away from the high-rise building. It is inferred that the influence of the high-rise building extended to several tens of meters away. For Sections I (inside old town) and J (south side of old town), $\bar{S↓}$ and $\bar{L↑}$ were high, and $\bar{T_{a}}$ was also high at 10:30 and 12:00. Although $\bar{L↑}$ was high throughout the afternoon, $\bar{T_{a}}$ was low at 17:00 and 19:00. The characteristics of daily variation of S↓, $\bar{L↑}$ (T_s_), and T_a_ for areas with shaded pavement (Section C) are different from that for sunlit areas with roofs having low heat capacity (Sections I and J).

## 5. Discussion

### 5.1. Formation Mechanism of Locally Low T_s_ and T_a_ in the Shade of a High-Rise Building

As mentioned in the Introduction, the formation mechanism of UCIs and lower T_a_ in deep canyons have been investigated [3,4,5,6,7,8,9]; however, locally lower T_a_ than T_a_′ in the shade of a high-rise building have not been observed, and formation mechanisms of the locally lower T_a_ have not been discussed. Causes for significantly lower T_s_ than T_a_′ and locally low T_a_ for a wide shaded area that is open to areas beyond the canopy (i.e., in front of the high-rise building and the adjacent commercial building) are discussed in this section. According to the remote sensing observation, areas where T_s_ is lower than T_a_′ by several degrees, are even observed in the daytime. A primary factor for the low T_s_ is atmospheric radiative cooling [18]: L↑ (T_s_) from mobile observations differed between areas under a large roof and areas outside of it (Figure 9). Figure 3a shows that a low T_s_ can be observed near buildings especially in front of the high-rise building and the adjacent commercial building (Section C), indicating the importance of long-term shade and cold storage of the surface material (i.e., pavement). According to the mobile observations, the amount of short-wave radiation was small in Section C throughout the day (Figure 9) (most part of the Section C is persistently shaded). Cold storage of the surface material can be described as follows: T_s_ of the pavement (Figure 3a(6)) was lower than that of other surrounding materials (short plants, soil, and roof shown in Figure 3a(3), (7), and (8), respectively) although the difference in the radiation budget (i.e., S↓ + L↓ in daytime and L↓ nighttime) between these materials was considered to be minimal. This fact indicates that the high heat capacity of the pavement contributed towards lowering T_s_ significantly. More specifically, the T_s_ of the pavement did not increase like other materials because the cooling heat from atmospheric radiation cooling at nighttime was stored owing to a high heat capacity.

The T_a_ in front of the high-rise building and the adjacent commercial building was lower than the T_a_ of its surroundings and T_a_′ in the daytime (Figure 9). According to Figure 9, at 10:30, Sections D and E were in the shade of the high-rise building, and S↓ was as low as the S↓ of section C. However, the T_a_ of Sections D and E was similar to that of the T_a_ of its surroundings and higher than the T_a_ of Section C by 1–2 °C. The L↑ of Sections D and E was also higher than the L↑ of Section C by about 10 W/m^2^. Therefore, it is suggested that the low T_a_ in front of the high-rise building and the adjacent commercial building is caused by the continuous generation of cold air by local heat exchange between the air in the vicinity of the surface and the cold surface, rather than the advection of cold air from the surrounding areas or the sinking of atmospheric cold air. A low L↑ and T_a_ can be observed in areas other than Section C, although L↑ and T_a_ are not as low as in Section C. Scatterplots of L↑ and T_a_ in Figure 10 clearly show that T_a_ is locally low in the areas having low L↑ (T_s_) in the daytime. It is also suggested that the interaction between cooled air in the vicinity of the surface and the cold surface is important for the formation of low T_s_. More specifically, a lower T_a_ in the vicinity of the surface yields a lower heat flux toward the surface. The low heat flux is considered to contribute to the persistence of low T_s_.

The principle of the generation of cold air by local heat exchange between the surface and the air in the vicinity is described as follows: the only cooling sources for the generation of cold air are the ground and wall surfaces where T_s_ is low. If there are no cooling sources, cold air is dispersed along the prevailing flow of air, resulting in no areas being formed where the T_a_ is locally low. It appears that the surface can continuously cool the air in the vicinity while T_s_ hardly increases since the heat capacity of ground and wall surfaces is −500 times higher than that of air. Even though the generated cold air is advected into the surrounding areas due to strong winds (Section C), a dynamic equilibrium condition in which T_a_ is locally low seems to be formed by the continuous generation of the cold air through convective heat transfer. This type of phenomenon is likely to occur, especially in large, shaded areas, due to long fetch (−50 m)-influenced advection.

### 5.2. Significance of Locally Low T_s_ and T_a_ in Urban Climate Studies

The importance of the finding of the microclimate in the area that is open to beyond the canopy in the context of urban climate studies is discussed in this section. Cold storage and lower T_a_ in the canyon than outside of it during daytime have been observed for closed canyons surrounded by buildings. In the case of a high-rise building, lower incoming solar radiation and lower T_s_ in the shade than those in sunlit areas are obvious. However, locally low T_a_ in the shade is not straightforward since the distribution of T_a_ is strongly affected by wind flow [19]. The influence of air inflow from and outflow to surrounding areas that reduce the locality of T_a_ is considered to be more significant than that for densely built central areas and deep canyons. Because in the case of a high-rise building and surrounding low-rise areas, certain spaces are open to areas beyond the canopy, and the extent of the shade is limited.

The results of the present study show that low-temperature environments, where T_a_ is lower than the surrounding T_a_, could be formed even at spaces open to areas beyond the canopy. Even for areas 70 m away from the high-rise building, shaded by the surrounding buildings and the high-rise building for most of daytime, T_a_ was low compared to other areas in the study site. Such local low-temperature environments seem to affect not only the outdoor thermal comfort but also the indoor thermal comfort and energy consumption of the houses. Numerical simulation is a feasible approach to assess the influence of a high-rise building on the surrounding thermal environment and energy consumption. Models that calculate T_a_ while considering the temporal variation of airflow and heat exchange between air and surface [20,21,22,23] are required to consider the cold storage and heat exchange discussed in the present study. In most cases, coupling between fluid dynamics calculation and heat balance calculation is limited to 1 day [20,21,22,23]. Long-term cold storage seems to contribute to the low T_s_ and T_a_ since the pavement in front of the high-rise building and the adjacent commercial building is persistently shaded. Therefore, it is possible that the coupling calculation must be improved so that the long-term effect is considered in the numerical simulation.

Concerning the relationship between the distribution of T_s_ and that of T_a_, thermal remote sensing has often been used. Many studies investigated the relationship between satellite-derived T_s_ and ground-based T_a_ [24,25,26,27,28,29,30]. The correlation between T_s_ and T_a_ differs according to the time of day, season, land cover, spatial geometry, and spatial resolution of T_s_ and T_a_ data. A few studies employed airborne remote sensing that can observe detailed T_s_ distributions in urban districts [31,32,33,34]. Coutts et al. [33] investigated the ability of airborne remote sensing to detect hot spots (areas with relatively high T_a_) in an urban area in summer using airborne T_s_ data with a spatial resolution of 0.5 m and T_a_ distribution by automobile observation at midnight. They concluded that high spatial resolution T_s_ data should be aggregated to a coarser resolution (>30 m) to detect hot spots. We showed that high spatial resolution T_s_ data was advantageous for the investigation of the distribution of T_a_ in urban areas with many winter daytime shaded areas, further suggesting an interaction between T_s_ and locally low T_a_ in shaded areas.

### 5.3. Limitations of the Present Study

The accuracy of remote sensing sensors, uncertainty of the emissivity correction (Appendix C), and accuracy of the sensors used in the mobile observations validate the significance of the aforementioned results and their associated discussions. Even if the observations exhibited a small bias, the relative variation (i.e., the difference in temperature) recorded within the study area was unaffected. A typical urban district that consists of a high-rise building and surrounding low-rise areas was selected, and observations were conducted in clear-sky days during winter. The observed significantly low T_s_ and T_a_ are not considered to occur under specific conditions. However, observations for one site on one day limit the discussion about occurrence frequency and occurrence conditions of low T_s_ and T_a_. The present results are insufficient to elucidate the detailed formation mechanisms of low T_s_ and T_a_, which are, possibly, complicated thermal and fluid phenomena. Therefore, multiple-point observations (horizontal and vertical) under various meteorological conditions and spatial geometries are required for future studies. Three-dimensional numerical simulation considering the spatial form and surface material of the urban canopy as well as heat and cold storage of the ground and wall surfaces, and the interaction between the surface and the atmosphere is a feasible approach for clarifying the influence of local heat exchange between the surface, the air in the vicinity, and local heat storage of the surface material on low T_s_ and T_a_ values.

There was a time interval of about 6 years between the airborne remote sensing and the mobile observations in the present study. Because of this limitation, we were not able to examine quantitatively the ability of airborne remote sensing to detect cool spots of T_a_ (quantitative relationship between remotely sensed T_s_ for each pixel and surrounding T_a_). Therefore, it was ideal that both observations were conducted simultaneously. However, influence of the time interval on the above-mentioned results and discussions was considered to be small since the results of remote sensing were not directly compared to those of mobile observations, and L↑ derived by the mobile observations showed characteristics similar to those derived by the airborne remote sensing. In addition, it is an effective and common approach in urban environmental researches that environmental phenomena are identified from data over wide areas provided by airborne remote sensing, and then, the phenomena are investigated deeply by terrestrial observations. Again, it is ideal that airborne and terrestrial observations are conducted simultaneously, especially for examining the ability of airborne remote sensing to detect cool spots as in [33]. Airborne remote sensing should be conducted simultaneously with detailed terrestrial observations mentioned in the former paragraph in future research.

## 6. Conclusions

Winter air temperature and the factors affecting it, i.e., surface temperature, wind velocity, and radiation budget, in low-rise areas around a high-rise building were investigated using airborne remote sensing data and terrestrial mobile observation data. According to remote sensing observations, the daytime T_s_ of pavements with a high heat capacity was significantly lower than T_a_ of the sub-urban area, indicating the influence of cold storage on T_s_. According to our mobile observations, S↓ and L↑ were low throughout the daytime regarding the front of the high-rise building and the adjacent commercial building, and T_a_ was low, especially from morning to noon. Although strong wind was present around the building, the T_a_ in front of the building was lower than that of sunlit areas in the same urban area by −2 °C and lower than that of sub-urban areas by −1–1.5 °C during daytime. The distribution of T_a_ corresponded well to that of L↑ in the daytime. Interaction between T_s_ and locally low T_a_ appears to be an important cause of the significantly low T_s_. The present study suggests that a significantly low T_s_ and a locally lower T_a_ than that of the surrounding areas during the day could develop even in spaces that are open to areas beyond the canopy, as in the case of the shade of an isolated high-rise building. This is a result of long-term shade and atmospheric radiation cooling, as well as the high heat capacity of relevant materials, and the interaction between T_s_ and local T_a_. This highlights the thermal environment problem, in addition to the blocking of sunlight, caused by a high-rise building in winter, during the day. The airborne and terrestrial data were analyzed carefully so that the conclusions were not affected by the time interval of about 6 years between the airborne remote sensing observation and the terrestrial mobile observations; however, the quantitative relationship between the airborne and terrestrial data could not be investigated because of the time interval. In future work, airborne remote sensing and mobile observations will be conducted simultaneously to further examine the ability of airborne remote sensing to detect cool spots of T_a_. In addition, we aim to conduct numerical simulations, in addition to remote sensing and in situ mobile observations, to elucidate the formation mechanisms of low T_s_ and T_a_ in more detail.

## Figures and Tables

**Table 1:** Specifications of remote sensing observations.

Items	Detailed Specifications
Dates	12:16, 22 December 2009 16:53, 22 December 2009
Altitude	500 m
Scanner	AZM (Nakanihon Air Service)
Scan Angle	80° (±40°)
Instantaneous Field of View	1.25 mrad
Number of Bands	0.40–0.85 μm: 5 bands 0.90–1.70 μm: 5 bands Thermal: 2 bands
Thermal Infrared Band Used	10.1–13.5 μm

**Table 2:** Items and devices used for mobile observations.

Items	Devices
Air temperature and relative humidity	Ø 0.1 mm T-type thermocouple and humidity sensor (CHS-UPS, TDK) installed in forced ventilation pipe
Wind velocity	Handheld hot-wire anemometer (Climomaster) Model 6501 series, KANOMAX (Osaka, Japan)
Downward and upward radiation (short-wave and long-wave)	Four component radiometer (MR-60, EKO Instruments (Tokyo, Japan), 0.285–3 μm for short-wave, 3–50 μm for long-wave)
Recording	Data logger (Thermic Model 2300A, ETO DENKI (Tokyo, Japan)

**Figure A1:**
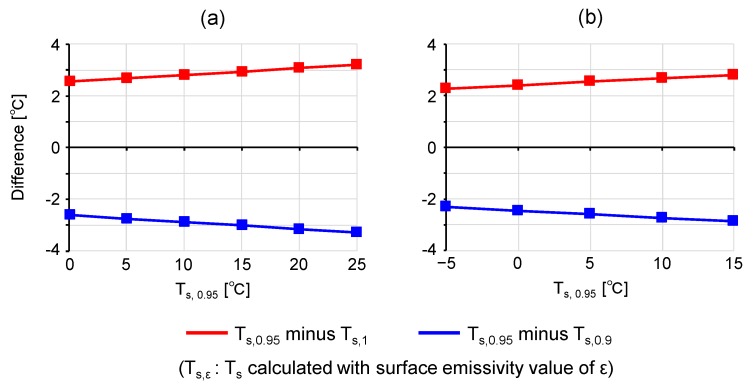
Difference between T_s_ calculated using emissivity value of 0.95 and that calculated using other emissivity values (1 and 0.9): (**a**) daytime and (**b**) after sunset. The difference for emissivity 1 is depicted with red line, and that for emissivity 0.9 is depicted with blue line. The difference indicates the uncertainty of T_s_ used in the present study (i.e., calculated using emissivity value of 0.95): T_s_ used in the present study was higher (lower) than the actual T_s_ if the actual emissivity of the surface was 1 (0.9).

## Appendix A. Geometric Correction

The ortho–correction process was conducted using a digital elevation model with a spatial resolution of 5 m provided by the Geospatial Information Authority of Japan (GSI). Subsequently, 100 ground control points were acquired for each image using a 1/2500 urban map of the geographic information system of the study site provided by GSI. A first-order affine transformation was then applied to correct the geometric positions. Ortho-correction using a digital surface model (building height) was not conducted, however this hardly affected the analysis in Section 3 because of following reasons. For Section 3.2.1 and Section 3.2.2, T_s_ for the specific points was manually extracted. For Section 3.2.3, when ortho-correction using a digital surface model (building height) is not conducted, the shape of the T_s_ histogram varies due to the difference in viewing angle (the viewing zenith angle differs between the northern area and the old town). We estimated that the fraction of the pixels of wall surfaces was about 5% for the northern area and 9% for the old town, based on the location, shape, and height of the buildings and the viewing geometry. Therefore, a difference in the shape of the T_s_ histogram between the two areas is barely explained by not conducting ortho-correction using digital surface model.

## Appendix B. Atmospheric Correction

In general, brightness temperature (T_b_) obtained by airborne observation differs from radiative surface temperature (T_r_) of ground surfaces due to the absorption and thermal radiation of molecules in the atmosphere. To obtain T_r_, the atmospheric effect was corrected as follows. T_r_ was observed at ground level using an infrared thermal camera (TH9100-MR, NIPPON AVIONICS Co., Ltd., Tokyo, Japan) at the same time as airborne observations. Materials with high, medium, and low T_r_ were observed, and the linear relationship between T_b_ and T_r_ was obtained [35]. Ground observation and correction were conducted separately for daytime and post-sunset measurements. Regarding daytime observations, asphalt pavement, sandy soil, and water were observed as the materials having high, medium, and low T_r_, respectively; after sunset, this changed to asphalt pavement, water, and grassland. For each material, observations were conducted at several points within a 2–3-m plot, and the obtained values were averaged. A correction equation was then obtained (Equations (A1) and (A2) for daytime and after sunset, respectively) and applied to all pixels.

T_r_ = 1.09 × T_b_ − 1.07 (A1)

T_r_ = 1.14 × T_b_ − 1.88 (A2)

The spectral range differs between remote sensing and infrared camera. The influence of this difference on the corrected T_r_ was estimated to be about 0.2 °C by using the below-mentioned radiative transfer model and by assuming spectrally constant sensor sensitivity. The difference is smaller than the estimated value when the actual sensitivity is considered due to a low sensitivity at the edge of spectral range.

## Appendix C. Emissivity Correction

When the directionality is assumed to be negligible, the relationship between T_r_ corrected for the atmospheric effect and T_s_ is expressed as follows:

L(T_r_) = εL(T_s_) + (1 − ε)L_in_, (A3)

where L(T) is the radiance of black body radiation at temperature T, ε is the surface emissivity, and L_in_ is the radiance of incident long-wave radiation on the surface. The radiances correspond to the wavelength range of remote sensing. To calculate T_s_ using Equation (A3), ε and L_in_ are needed. Several methods have been developed for retrieving surface emissivity from satellite data, as summarized in Li et al. [36]. For high-resolution remote sensing of urban areas, ε and L_in_ differ from pixel to pixel. One possible method for estimating ε is to conduct landcover classification and to assign ε values from the data base. However, accurate landcover classification is difficult for images with many shaded areas. It is also difficult to obtain L_in_ for each pixel. Therefore, constant values of ε and L_in_ were used. ε was set to 0.95 based on studies that report ε values for materials occupying most of the urban surfaces, including concrete, asphalt, slate roof, clay roof tile, soil, water, and vegetation [37,38,39,40,41].

For L_in_, we used downward long-wave radiation measured at the JMA Aerological Observatory located 7.5 km west of the study site (36°3′ N, 140°7′ E). The irradiance of downward long-wave radiation is measured on a horizontal surface with a sampling interval of 1 s, and the hourly mean irradiance is available on the JMA website. We used the hourly mean irradiance value for the period during which the remote sensing observation was conducted, based on the assumption that the downward long-wave radiation varied little over 1 h. The wavelength range of the measurements was 4–40 μm and is different from that of remote sensing. Therefore, to make the conversion, the ratio between the radiance for the wavelength range of the Observatory measurements and that of remote sensing was calculated using an atmospheric radiative transfer model (MODTRAN 5, developed by Spectral Science, Inc. and US Air Force Research Laboratory) [42,43]. Parameters to run the model included the vertical distributions of T_a_, air pressure (P), density of aerosols, and gasses (H_2_O, CO_2_, O_3_, N_2_O, CO, and CH_4_). For T_a_, P, and H_2_O, the vertical distributions measured by the Aerological Observatory were used. The observatory measures the vertical distribution of T_a_, V, Rh, and P twice a day at 09:00 and 21:00 local time. The vertical distribution measured at 09:00 and 21:00 were used for the input data for remote sensing observation during daytime and post-sunset, respectively. For aerosols, the default rural extinction model [42,43] with a meteorological range of 23 km was used, according to the observed visibility at the observatory. A mixing ratio of 380 ppm was used for CO_2_. For other gases, the default Mid-Latitude model [42,43] was used. T_s_ was then calculated using Equation (A3).

To evaluate the uncertainty of T_s_, the differences between T_s_ calculated with an emissivity of 0.95 and that calculated with emissivity values of 1 and 0.9 were obtained (Figure A1). Figure A1 indicates that the remotely sensed T_s_ (Figure 3) overestimated and underestimated the temperature of surfaces having emissivity values of 1 and 0.9, respectively, by 2.5–3.5 °C. The Aerological Observatory measures downward long-wave radiation at an open space (the sky view factor is almost 1). Therefore, L_in_ is underestimated especially for ground surfaces since it includes radiation from surrounding buildings. The calculated T_s_ becomes lower when a larger L_in_ value is used (e.g., the calculated T_s_ becomes lower by −1 °C when L_in_ is calculated under the assumption that the sky view factor is 0.7 with building surfaces having T_s_ of T_a_′ + 10 °C).
